# The gut-brain axis in ischemic stroke: its relevance in pathology and as a therapeutic target

**DOI:** 10.1186/s42466-022-00222-8

**Published:** 2022-11-14

**Authors:** Corinne Benakis, Arthur Liesz

**Affiliations:** 1 Institute for Stroke and Dementia Research (ISD), University Hospital, LMU Munich, Munich, Germany; 2 Munich Cluster for Systems Neurology (SyNergy), Munich, Germany

**Keywords:** Stroke, Brain ischemia, Microbiota, Gut, Inflammation, Metabolites

## Abstract

The gut contains the largest reservoir of microorganisms of the human body, termed as the gut microbiota which emerges as a key pathophysiological factor in health and disease. The gut microbiota has been demonstrated to influence various brain functions along the “gut-brain axis”. Stroke leads to intestinal dysmotility and leakiness of the intestinal barrier which are associated with change of the gut microbiota composition and its interaction with the human host. Growing evidence over the past decade has demonstrated an important role of these post-stroke changes along the gut-brain axis to contribute to stroke pathology and be potentially druggable targets for future therapies. The impact of the gut microbiota on brain health and repair after stroke might be attributed to the diverse functions of gut bacteria in producing neuroactive compounds, modulating the host’s metabolism and immune status. Therefore, a better understanding on the gut-brain axis after stroke and its integration in a broader concept of stroke pathology could open up new avenues for stroke therapy. Here, we discuss current concepts from preclinical models and human studies on the bi-directional communication along the microbiota-gut-brain axis in stroke.

## Background and introduction

Stroke is the second largest cause of death after ischemic heart disease worldwide, with ischemic stroke accounting for 60–85% of cases depending on regional epidemiology [1, 2, 3]. Currently, thrombolysis with recombinant tissue plasminogen activator (rt-PA) and endovascular thrombectomy given in the hyperacute phase after ischemic stroke onset are still the only effective therapies [4, 5]. Due to the narrow therapeutic time window and safety concerns, the clinical indications for thrombolysis and mechanical thrombectomy are limited and most stroke patients do not receive a specific acute stroke treatment [6].

Circulating humoral and cellular factors, including immune cells, metabolites and cytokines/chemokines have been increasingly recognized to play a critical role in stroke pathophysiology [7, 8]. Therefore, such circulating mediators and immune cells between peripheral organs and the ischemic brain have received growing attention as promising therapeutic targets for stroke treatment.

In this regard, the gut microbiota—the highly complex communities of microorganisms that inhabit the intestinal tract—has become a focus of biomedical research, as the gut microbiota is a critical superorganism that regulates many physiological functions of the host. The homeostasis of the gut microbiota affects not only the gastrointestinal tract (GIT) environment, but also distant organs, including the brain [9]. Early colonization of the gut microbiome is crucial for brain function and behavior, as its absence results in blood–brain barrier (BBB) damage, altered synaptic plasticity, and deficits in social behavior [10]. Germ-free (GF) mice also exhibit an immature microglia phenotype, leading to reduced immune responses [11]. The bidirectional communication between gut and brain suggests a key role for the gut microbiota through regulation of host metabolism, immune system and vascular function [12]. In addition, the gut microbiota can also influence the central nervous system (CNS) via the vagus nerve (VN) by transmitting signals from the gut microbiota to the brain and vice versa, both in health and disease [13], including stroke [14, 15]. In this review, we focus on the role of the gut microbiota in ischemic stroke and discuss the current state of knowledge on the impact of the microbiota on disease outcome and the potential mechanisms involved in these interactions.

## Changes of the gut microbiota composition after stroke

Several preclinical and human studies have demonstrated changes in the gut microbiota composition after ischemic stroke [16, 17, 18]. Among these changes, high-level associations such as an increase in members of Enterobacteriaceae and a decrease in SCFA-producing bacteria have been consistently shown by several groups [18, 19, 20]. Despite this, evidence of the involvement of specific types of bacteria or metabolites produced by the microbiota have not yet been identified.

We have previously demonstrated in a mouse stroke model a reduction in species diversity and overgrowth of bacteria of the genus Bacteroidetes in the gut [17]. Another study found that in the intestine of cynomolgus monkeys, the concentrations of the genera Bacteroidetes and Prevotella increased significantly after inducing experimental stroke, while the concentrations of the genera Firmicutes and Faecalibacterium decreased [21].

Analysis of gut microbiota composition in stroke patients confirmed the shift in microbial communities, with a correlation between stroke severity and the degree of gut microbiota changes [19, 22, 23]. Similarly, in mice, the changes in the microbiota composition is more pronounced after severe in comparison to a minor ischemic brain injury [17, 23]. However, when analyzing bacterial diversity and specific bacteria taxa, substantial differences can be observed between clinical and experimental studies. Importantly, studies reported an increase in diversity in stool samples from stroke patients in comparison to asymptomatic controls [23] or no change in microbiota diversity between sham operated and stroke mice [24]. Others observed a reduced diversity with loss of certain bacterial taxa and overgrowth of others in both experimental models and in patients [19, 22] suggesting that the degree of diversity does not correlate with the stroke severity. Other studies observed an overall reduction of the Firmicutes, with a concomitant overgrowth of Bacteroidetes [17, 25]. However, when analyzing microbiota changes at lower taxonomic ranks, there is a lack of consistency regarding the specific bacterial changes in clinical and experimental stroke [26, 27].

Alterations in the gut microbiota composition has been associated with the other diseases that present risk factors for ischemic stroke. Significant microbial perturbations have been observed in inflammatory bowel diseases (including Crohn’s disease and ulcerative colitis) which in turn resembles a relevant risk factor for ischemic stroke [28, 29]. In addition, the composition of gut bacteria in a high-risk population for stroke and cardiovascular disease also differs from that of the general population. Compared to the low stroke risk group, higher concentrations of opportunistic pathogens were found in the high-risk group, with Enterobacteriaceae being the most notable difference. Patients of the high-risk group had decreased levels of butyric acid-producing bacteria such as Lachnospiraceae and Ruminococcaceae [30]. An other study showed association with specific gut microbiota taxa and an increased risk of stroke-associated pneumonia [31]. These findings may suggest that disturbances in gut microbial homeostasis are associated with an increased risk of ischemic stroke. Therefore, analysis of gut microbiota composition could be an additional, and pathophysiologically relevant, biomarker for stroke prediction which should be further investigated in future studies on stroke risk factors. However, clinical data on the association between microbiota composition and stroke risk is rather sparse and does not provide a direct directionality or causality. Therefore, further prospective observational studies in high cardiovascular risk cohorts are required to test the potential predictive value of microbiota composition for cerebrovascular events.

The gut microbiota also varies among stroke patients in different age groups. The incidence of stroke is closely related to age, with approximately 70–80% of ischemic strokes occurring in people older than 65 years [32] and age plays an important role in stroke incidence and prognosis [33, 34]. The composition of the gut microbiota can be influenced by the environment, diseases and dietary habits, as well as age and gender differences [35]. The composition of the gut microbiota changes with age and its diversity decreases. When the gut microbial health is affected by environmental factors or aging, it can negatively affect physiological functions of the host and is thought to have an impact on age-related neurodegenerative diseases such as Alzheimer’s disease, Parkinson’s disease, and Huntington’s disease [26, 36, 37]. Bacteroidetes and Firmicutes predominate in the gut microbiota of young and elderly people. During aging, the relative abundance of Firmicutes increases, SCFA-producing bacteria and butyric acid levels decrease [38, 39]; correspondingly, permeability across the intestinal barrier is significantly higher in older adults [40]. A preclinical study in mice found that stroke outcomes in aged mice could be improved by transplanting the microbiota of young mice. Conversely, after acquiring the microbiome of aged mice, younger mice showed increased dysfunction after stroke [25, 40]. Furthermore, alteration of the gut microbiota associated with age was shown to be an independent risk factor for post-stroke infection, with older mice having a higher incidence and severity of post-stroke lung infection [41, 43]. This was related to a reduction of intestinal barrier integrity, translocation of intestinal bacteria into peripheral tissues and accompanied by a higher levels of circulating pro-inflammatory cytokines in aged mice in comparison to younger mice [25, 41–43]. Stroke-associated with lung infection was suggested to be the consequence of bacteria dissemination from the gut to the lungs [44]. It remains to be defined whether the gut microbiota is involved in worse outcome in elderly stroke patients and the development of post-stroke lung infection.

Additionally, sex is an important factor influencing the microbiota and post-stroke changes in bacterial composition. Men and women have been demonstrated in several studies to differ in the abundance of common bacterial taxa, such as Bacteroidetes [45]. There are also differences between the sexes in terms of post-stroke outcomes. Several studies have shown that women recover better than men after stroke [46]. Yet, in elderly patients, women have a significantly worse prognosis than men. This suggests that estrogen may play a protective role in women in the development of stroke. However, sex differences on microbiota aging might also play a crucial role in this phenomenon and consecutive effects on the expression of bacterial metabolites after stroke. Indeed, fecal butyric acid levels were significantly lower in men than in women after stroke [47], but LPS levels were found to be higher in men. In experimental stroke models, gut permeability was higher in male mice, suggesting that the male might by more susceptible to bacterial translocation and potential infection with gut-derived bacteria after stroke [47, 48].

## Mediators along the gut-brain-axis after stroke

### T cells

In permanent contact with the microbiome, epithelial and immune cells in the gut have evolved their capacity to maintain a homeostatic state by defending host integrity while promoting tolerogenic responses to commensal microbes. Under healthy conditions, pro- and anti-inflammatory mechanisms balance each other to preserve tissue homeostasis [49].

**Fig. 1:**
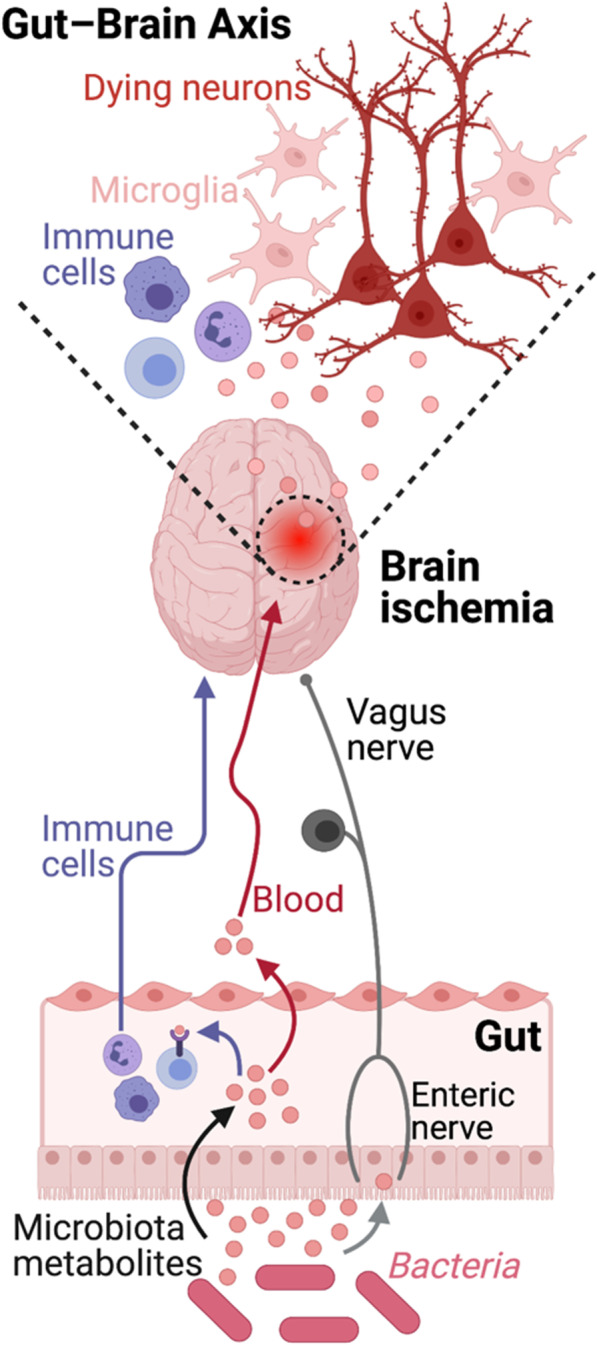
Microbiome-Gut-Brain axis in stroke. Current concept of the main pathways along the bidirectional gut-brain interaction. Here depicted are the 3 main pathways of brain to gut communication: (1) immune cells via the blood circulation, (2) vagus nerve axis and (3) systemically circulating bacterial metabolites released by the intestinal microbiota

T lymphocytes play a crucial role in stroke progression. In particular the pro-inflammatory lymphocyte γδT-IL-17+ cells have been shown to promote lesion development, whereas the anti-inflammatory T_reg_ cell population is mainly neuroprotective and involved in tissue recovery in the chronic phase [50, 51]. The dysbiotic microbiome was associated with an increase of CD4+-IL-17 cells in the gut and IL-17 expression in the brain [17]. Additionally, colonization of GF mice with a microbiome from conventionally housed mice was neuroprotective and showed a T cell response in the gut and in the brain, suggesting that mice with a conventional microbiome generate an adequate lymphocyte- driven immune reaction in response to brain injury and trigger tissue protection. Importantly, the microbiome-mediated brain protection was absent in lymphocyte-deficient mice [52]. Interestingly, it was highlighted that environmental factors modulating the gut microbiome are also important in mounting an inflammatory response in the gut. Indeed, mice from diverse commercial breeders have a substantial variation in their microbiota composition and this influences the intestinal T cell response and the impact on stroke [53]. In an other study, the key role of immune cell educated by the gut microbiome has been shown to have a considerable impact on stroke otucome. Bacterial priming of dendritic cells resulted in an expansion of regulatory T cells (T_reg_) in the gut, which secrete IL-10 to suppress the frequency of the pro-inflammatory IL-17+ γδT cells. Although this modulation of immune cells by the microbiota occurs in the gut, its effects were relayed to the brain, through T cell migration from the gut to the meninges [54]. These findings highlight a direct connection along the gut-brain axis via intestinal T cells regulating the neuroinflammatory response to stroke (Fig. 1).

### Vagus nerve

Previous topical reviews have described in detail the role of the autonomous nervous system in brain-body connection after stroke, e.g. [55]; here we want to focus instead on the afferent function of the vagus nerve as a well-characterized link between gut microbiome and brain. The vagus nerve (VN) provides bi-directional neuronal “hardwiring” and is a privileged mediator of the microbiome’s effect on brain function. This nerve regulates gut motility, secretions and inflammatory responses [56]. Studies in rodents demonstrate that following lipopolysaccharide (LPS) injection, VN releases acetylcholine in the gut and suppresses secretion of TNF-α by gut resident macrophages. Regulation of inflammation by the efferent VN is both local and systemic [57]. A functional role of the VN has been mainly studied in primary neurodegenerative disorders with clinical relevance, particularly in Parkinson’s disease. Several studies using vagotomy to study the role of the VN have suggested its causal role in Parkinson’s disease pathophysiology as a link between the gut microbiome environment and the cerebral pathology. In ischemic stroke, the vagus nerve has been widely studied, both for its afferent and efferent function, to modulate neuroinflammation and neuronal plasticity after stroke [58, 59]. Stimulation of the vagus nerve has been recently shown to ameliorate motor function in stroke patients [60]. However, in contrast to neurodegenerative disorders, the function of the VN to mediate changes in the gut microbiome and the recovering brain after stroke has been barely studied [61, 62]. It is unknown whether the clinically proven efficacy of VN stimulation [60] would be (partially) mediated via changes in the gut microbiota composition to improve post-stroke recovery. Indeed, this clinical study did not analyze effects on gut microbiota composition between the intervention groups and potential effects of direct VN stimulation on the gut microbiome which will require analysis in future studies. Therefore, despite convincing findings demonstrating per se for a potential involvement of the VN also in acute [63] brain diseases including stroke, its role in linking the gut microbiome to specific stroke pathophysiology requires further investigation.

### Microbial metabolites

Small molecules from the gut microbiome form the molecular basis of host-microbiome interactions. Some of these molecules, bacterial metabolites, have been shown to affect host physiology and play an important role in stroke pathology (Fig. 1).

#### Short-chain fatty acids

When intestinal bacteria break down dietary fiber, large amounts of short-chain fatty acids (SCFA) are produced in the gut: acetate, propionate and butyrate. SCFAs improve intestinal motility, reduce inflammatory cytokines, and regulate adaptive immune tolerance and levels of gut hormones and neuropeptides [64]. SCFAs have an immunomodulatory function and induce differentiation of T cells into effector or regulatory cells depending on the immunological environment [65]. Furthermore, SCFAs are directly linked to brain function; for example, monocolonization of butyrate-producing bacteria restores BBB integrity and plays a key role in microglial maturation [11]. Recently, we have shown that SCFA supplementation in mice prior to stroke improves behavioral recovery, alters cortical network connectivity, and modifies histological markers of synaptic plasticity, which is associated with improved long-term stroke outcomes [66]. These effects were associated with changes in microglial morphology toward a homogenized transcriptome signature. This study showed for the first time that gut SCFA metabolites are involved in post-stroke recovery [66]. Another recent study in aged mice showed that SCFA levels decrease with aging and contribute at least in part to poorer stroke outcome in aged mice [40]. Therapeutic administration of probiotic inulin and SCFA-producing bacteria to elderly mice after stroke significantly improved stroke prognosis. Also in this study, a significant reduction of IL-17 + γδ T cells was observed in the brains of aged mice treated with SCFA-producing bacteria compared to vehicle-treated controls. This may suggest that SCFAs reduce inflammation induced by IL-17 + γδ T cells after stroke [40]. In both studies, SCFAs appeared to reduce the frequency of pro-inflammatory T cells in the brain after stroke, but whether SCFAs directly affect T cell polarization and migration remains to be fully elucidated.

#### Tryptophan metabolites

Tryptophan is an essential amino acid sourced from the diet that can be metabolized via three different pathways: the kynurenine pathway, mainly by immune cells and epithelial cells, the serotonin pathway by enterochromaffin cells and the indole pathway by the gut microbiota [67]. The kynurenine pathway accounts for more than 90% of tryptophan metabolism and is implicated in a number of neurodegenerative diseases [68] and acute brain disorders. Several studies have shown that increased tryptophan catabolism via the kynurenine pathway is positively correlated with stroke severity in patients and may be associated with stroke-induced inflammation [69, 70].

The indole breakdown products of tryptophan are produced by several bacterial species and act as important immunomodulators [71, 72]. Indole metabolites bind to the aryl hydrocarbon receptor (AHR), a transcription factor originally shown to act as detoxicant and recently involved in modulating the immune response. AHR is expressed in various immune cells of the innate and adaptive immune system, including pro-inflammatory TH17, anti-inflammatory T_reg_ cells, dendritic cells and brain resident glial cells [73, 74, 75]. AHR-deficiency has been shown to improve experimental stroke outcome, associated with reduced neuroinflammation and increased neuronal differentiation [76]. Hence, tryptophan degradation products from microbiota may be interesting targets for modulating immunological and neuronal responses to brain injury.

#### Bile acids

Bile acids are synthetized in the liver from cholesterol and secreted into the intestinal tract to facilitate the digestion and absorption of dietary lipids. Once in the small intestine the two main primary bile acids cholic acid and chenodeoxycholic acid are further metabolized by the intestinal microbiota to produce secondary bile acids.

Both primary and secondary bile acids have roles beyond their lipid detergents properties, and can function as potent immunomodulators [77]. The bile acids-receptors farnesoid X nuclear receptor (FXR) and G protein-coupled bile acid receptor 1 (GPBAR1 or Takeda G-protein receptor 5) are expressed by different immune cells regulating the balance between immunity and tolerance. For example, FXR-deficient macrophages are polarized towards a pro-inflammatory phenotype at homeostatic states and secrete increased levels of pro-inflammatory cytokines [78, 79]. Bile acids are also capable of crossing the blood–brain barrier and interacting directly with local brain cells. Previous reports have demonstrated a direct effect on neuronal function by circulating bile acids [80]. In experimental stoke models, intravenous administration of the synthetic tauroursodeoxycholic acid (TUDCA) is neuroprotective, improves neurologic functions and reduces the infarct size [81]. Correspondingly, TUDCA was also effective in a traumatic brain injury model to improve outcome by reducing apoptotic neuronal cell death [82]. Moreover, treatment with TUDCA was associated with anti-inflammatory effects in a model of neuroinflammation, resulting in reduced glial activation and migratory capacity [83]. In summary, bile acid modulation through the gut microbiota represents an attractive therapeutic option for the treatment of brain injury since they exert anti-inflammatory activity, lessen neuronal cell death, as well as inhibit monocyte trafficking and induce tolerogenic T cells in the gut [84, 85].

## Therapeutic approaches to modulate the gut microbiome after stroke

### Dietary interventions

Dietary regulation is an important measure to improve the prognosis of stroke and has emerged as an established component of life-style interventions in order to reduce neurovascular risk [86]. Most clinical guidelines recommend a diet that reduces saturated fat and cholesterol intake and an increase in fruit and vegetable diet as a source of increased fiber intake; this can increase the level of SCFAs production by the gut microbiota which use fiber as a fermentation source. For example, resistant starches (such as whole grains and legumes) and fructo-oligosaccharides as metabolic food source can increase butyric acid production in the gut [87].

Energy control is an effective way to promote good health and reshape the intestinal symbiotic microbiome. Some studies suggest that energy restriction to 60–70% of the recommended intake is protective against ischemic stroke [88]. The protective effect of energy control on brain injury after stroke may be realized by promoting glycogen metabolism and adiponectin expression [89, 90]. Caloric restriction resulted in significant changes in the composition of the gut microbiota, with a specific enrichment of Bifidobacteria, which was associated with improved functional outcome in mice subjected to ischemic stroke [91].

### Probiotics and prebiotics

Probiotics are a group of living gut microorganisms that are widely believed to be beneficial to the host. Probiotics may protect tissue from damage by alteration of tissue homeostasis, for example by reduction of oxygen free radical production. As such, probiotics can inhibit the production of pro-inflammatory cytokines including TNF-α in vivo, promote the generation of anti-inflammatory cytokines, and increase anti-oxidant pathways [92]. Previous studies have demonstrated significant reduction in stroke severity in mice with focal brain ischemia which received supplementation with probiotics mainly from the Bifidobacterium and Lactobacillus taxa [93]. Pretreatment with Clostridium butyricum was shown to effectively inhibit apoptosis in a rat stroke model, resulting in improved functional outcome [94]. In addition, regular consumption of lactobacillus probiotics can also alter the expression of brain-derived neurotrophic factor (BDNF) receptors and increase BDNF concentrations in the brain, which can promote recovery after brain ischemia [95].

The most common prebiotics are oligosaccharides—known to have no direct biological activity, being non-digestible and degraded by the gut microbiota—such as lactulose oligosaccharide, isomaltose oligosaccharide, fructose-oligosaccharide, lactulose oligosaccharide and inulin. After entering the lower intestinal tract, prebiotics can be hydrolyzed and used as nutrients by diverse microbiota, hence, promoting the growth and diversity of these bacteria, particularly metabolically more demanding bacteria. In addition, prebiotics can regulate intestinal homeostasis by increased production of SCFAs and regulation of mucin production, which in turn affects function of intestinal immune cells [96]. In one meta-analysis, probiotics/prebiotics effectively reduced the incidence and severity of pneumonia during hospitalization in critically ill patients [97]. These findings could suggest that the use of probiotics/prebiotics could play a disease-modulating role in severely ill stroke patients with impaired microbiota composition and intestinal function.

### Fecal microbiome transplantation

The transfer of the entire gut microbiota from the stool of a healthy donor to the patient’s intestinal tract is known as fecal microbiome transplantation (FMT). The technique is already being used to treat patients with some forms of severe bacterial infections mainly of the intestinal tract [98]. In addition, FMT intervention has been shown to potentially relieve symptoms in patients with Parkinson’s disease and reduce autism in children with autism disorder [99]. However, because the gut microbiota also may cause disease, e.g. by transfer of potentially harmful bacteria to the specific recipient host, it is important to select suitable healthy FMT donors. Transplantation of feces which was either selected or enriched to contain potentially beneficial bacterial taxa (e.g. SCFA-producing bacteria) has been shown to regulate the composition of intestinal microbes, resulting in improved intestinal wall integrity and reduced intestinal leakage in rats subjected to ischemic stroke [100]. In addition, transplantation of the fecal microbiome from young mice to aged animals that were subjected to experimental stroke improved stroke outcome [25]. Similarly, transplantation of feces which was enriched in butyrate-producing bacteria reduced ischemic stroke injury in diabetic mice [101]. Whereas there is no information yet on the beneficial effect of FMT in stroke patients, a recent study performed FMT of patients with post-stroke cognitive impairments (PSCI) into mouse recipients subjected to stroke [102]. They were able to recapitulate the pro-inflammatory profile of PSCI patients which was associated with cognitive deficits, an increase in brain barrier permeability and hippocampal neuronal death in the mouse recipients. These deleterious effects were reversed by the supplementation of the SCFA sodium butyrate [102].

### Use of antibiotics in stroke patients

Approximately 30% of stroke patients experience a bacterial infection within 1 week of incident stroke [103], therefore a significant number of patients receive early anti-infective therapy with antibiotics, which often include a combination of broad-spectrum antibacterial drugs, despite often a lack of proven bacterial infection but based on a clinical criteria. However, clinical trials on the prophylactic use of antibiotics in stroke patients did not demonstrate a benefit for patient outcome [104]. Compared with standard treatment regimen, prophylactic use of antibiotics in stroke patients did not improve the long-term neurological status or mortality and had no significant effects on the incidence of post-stroke complications such as pneumonia. Moreover, a surprisingly large fraction of stroke patients are falsely diagnosed with supposed infection in case of only a sterile inflammatory immune response to the cerebral tissue injury without pathogenic infection [103, 105, 106, 107, 108]. Further studies have shown that extensive alteration of the gut microbiome by untargeted use of broad-spectrum antibiotics prior inducing ischemic stroke can substantially affect stroke outcomes being either beneficial or detrimental [54, 109]. When single antibiotic administration was used, ampicillin or vancomycin showed neuroprotective effect but not for the antibiotic compound neomycin which aggravated stroke outcomes [54, 110]. These different effects may be related to changes in the composition of intestinal flora or due to direct neurotoxic/-protective effects of the antibiotics. Importantly, acute treatment of mice with an antibiotic cocktail failed to improve stroke outcome [110]. Therefore, further work is needed to explore whether specific antibiotics can have a beneficial effect on the prognosis of patients with ischemic stroke and use this information to guide more targeted and individualized use of antibiotics in stroke patients.

## Conclusion and outlook

While the gut microbiota has received much attention in the context of immunology, metabolomic disorder and other brain diseases such as Alzheimer’s disease and Parkinson’s disease, its intricate function in the pathophysiology of ischemic stroke is just starting to be broadly recognized with first reports on this topic starting only in 2015 [23, 111]. Several preclinical studies over the past years and first clinical observational trials have described changes in the gut microbiota composition after stroke and linked these changes to physiological changes which might affect acute stroke outcome and even more so the chronic recovery after stroke. These changes along the so-called “gut-brain-axis” include major effects on the immune response to stroke, but also potentially direct neuromodulatory effects by soluble mediators (metabolites) produced by the gut bacteria or via the afferent function of the vagus nerve.

### Open questions

**Fig. 2:**
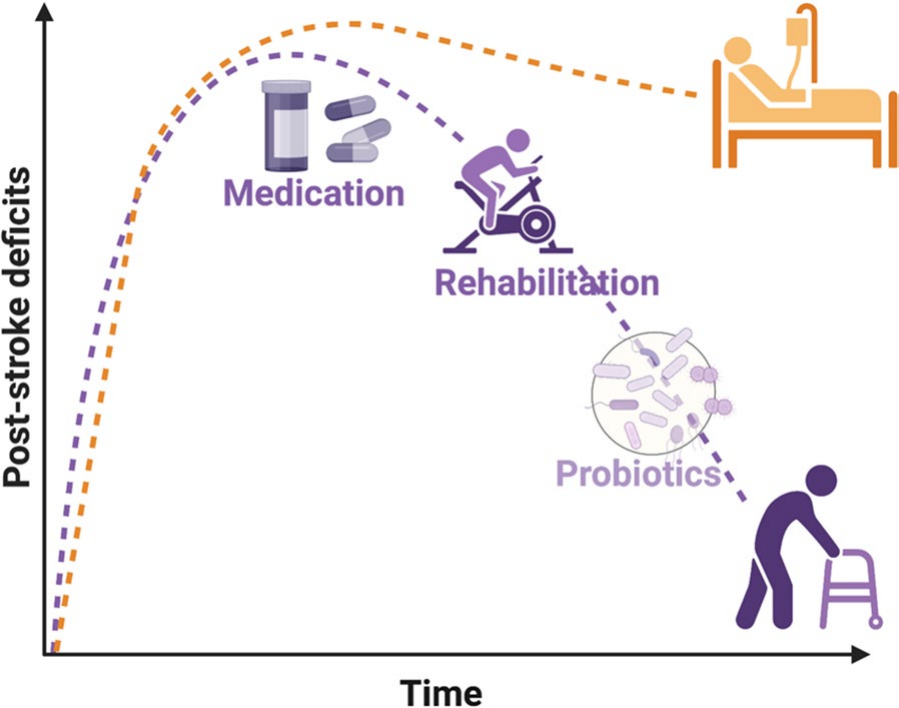
Potential use of microbiota-targeted therapies for post-stroke recovery. The scheme highlights the potential use of microbiota-targeted therapies such as probiotic therapies as supplementary therapeutic strategies to improve recovery during the chronic phase after stroke. Despite promising preclinical results and first clinical trials, current data is not yet sufficient to prove efficacy of such interventions and to suggest specific compounds or treatment regimens

While a modulatory function of the gut microbiota in the stroke pathophysiology is by now unequivocally demonstrated in several independent studies, key open questions on the mechanisms of microbiota-brain interaction remain to be answered. These include, but are not limited to, the question on the exact mechanism of the microbiota-host interaction. While multiple modes of interaction have been discussed and some have been studied in more detail also in the context of stroke (see above sections), a more detailed understanding on how changes of microbiota composition are able to change the remote inflammatory response in the brain and the regenerative capacity of the brain after stroke is yet to be elaborated. Also the potential long-term consequences of stroke on gut and microbial function are still insufficiently described; yet, a better knowledge of the gut-brain interaction in the chronic phase might be of particular interest to further study and develop microbiota-targeted interventions as a supplementary therapy for post-stroke rehabilitation (Fig. 2).

### Clinical intervention trials

Clinical studies on the gut microbiota in stroke have so far been mainly observational in that they described changes in microbiota composition in the acute phase after stroke onset and its correlation with clinical parameters and prognostic factors. Only few interventional trials using probiotics have so far been reported, all of these studies have been performed in China with small population sizes each, different probiotic composition and only very limited information on the treatment efficacy. Nevertheless, a recent meta-analysis of these so far published interventional studies has observed a significant reduction in hospitalization days after stroke but no effect on NIHSS stroke severity [112]. Therefore, more studies with larger cohorts, improved study design and mechanistic information on the achieved effects on microbiota composition, immunity and functional outcome are warranted. Currently, four such studies are registered as recruiting in Clinicaltrials.gov (NCT05477732, NCT04978701, NCT03812445 and NCT04954846), of which 3 are performed at Chinese centers and one at our site in Germany. Considering the large impact of diet and geographical location on the gut microbiota composition, more studies in western populations will be required to shed light on the clinical relevance of microbiota interventions for stroke outcome across diverse populations, ethnicities and differences in dietary preferences.

### Potential for future translation

Despite the currently still limited mechanistic information on the role of the gut microbiome in stroke pathophysiology [113] and the above-discussed limitations of current clinical studies testing microbiota-targeted interventions, we assume that interventions using pre-/probiotic compounds could potentially be efficacious to improve stroke outcome or used as part of secondary prevention therapies.

As we discussed in more detail above, most studies show substantial changes in the microbiota composition, but also blood metabolism in the acute phase after stroke, which might be pathological response to tissue injury or (mal-)adaptive response. The time course and mechanisms for recovery to homeostatic microbiome function is not well characterized yet, but most likely represents a longer process over weeks and months after stroke.

Correspondingly, the use of probiotics was shown in previous studies to have only a minor to moderate impact on microbiota composition and effects on host physiology was achieved only after prolonged treatment periods [114, 115].

Therefore, we speculate that the most likely application of pre-/probiotic use will (if at all) not be for modulating acute stroke outcome but for affecting long-term outcome in the chronic recovery phase after stroke as previous experimental and clinical studies have demonstrated a potentially beneficial function of microbiota-derived metabolites on post-stroke recovery [20, 66]. Additionally, microbiota has been implicated in cardiovascular health and treatment of atherosclerosis [116]. Therefore, microbiota-targeted interventions including probiotics might also be potentially beneficial also as an add-on therapy in secondary prevention strategies.

The already broad experience in safe use of probiotic formulations make such therapeutic interventions despite probably only relatively minor effect a most likely safe approach as part of a general dietary intervention in stroke patients. Yet, the efficient clinical use of probiotics or other microbiota-targeted therapies in stroke therapy and secondary prevention is currently still speculative and requires prospective clinical intervention trials to demonstrate their efficient and safe use.
